# Determinant of factors associated with child health outcomes and service utilization in Ghana: multiple indicator cluster survey conducted in 2011

**DOI:** 10.1186/2049-3258-72-42

**Published:** 2014-12-01

**Authors:** Duah Dwumoh, Edward Eyipe Essuman, Seth Kwaku Afagbedzi

**Affiliations:** Department of Biostatistics, School of Public Health University of Ghana, Legon, Ghana; Department of Biological Environmental and Occupational Health Sciences, School of Public Health, University of Ghana, Legon, Ghana

**Keywords:** National health insurance, Under five, Multivariate analysis, Binary Logistic Regression, Hierarchical backward selection, Hosmer-Lemeshow Goodness-Of-Fit (HL-GOF), Akaike Information Criterion (AIC), Taylor linearization estimation, Chi-square test statistic, Enumeration areas, Anemia, Stunted growth and full immunization

## Abstract

**Background:**

The effects of National Health Insurance Scheme in Ghana and its impact on child health outcome and service utilization cannot be underestimated. Despite the tremendous improvement in child health care in Ghana, there are still some challenges in relation to how National health insurance membership, socioeconomic status and other demographic factors impacts on child health outcomes. The study seeks to determine the association between NHIS membership, socio-economic status, geographic location and other relevant background factors, on child health service utilization and outcomes.

**Methods:**

Secondary data from the Multiple Indicator Cluster Survey conducted in 2011 was used. Multivariate analysis based on Binary Logistic Regression Models and Multiple linear regression techniques was applied to determine factors associated with child health outcomes and service utilization. Collection of best models was based on Hosmer-Lemeshow Goodness-Of-Fit as one criterion of fit and the Akaike Information Criterion.

**Results:**

Controlling for confounding effect of socioeconomic status, age of the child, mothers education level and geographic location, the odds of a child developing anemia for children with National Health Insurance Scheme Membership is 65.2% [95% CI: 52.9-80.2] times less than children without National Health Insurance Scheme Membership. The odds of being fully immunized against common childhood illnesses for children with NHIS membership is 2.3[95% CI: 1.4-3.7] times higher than children without National Health Insurance Scheme Membership. There was no association between National Health Insurance Scheme Membership and stunted growth in children.

**Conclusions:**

National Health Insurance Scheme Membership was found to be related to child health service utilization (full immunization) of children under five a child’s anemia status. Children with NHIS are more likely to be fully immunized against common childhood diseases and are less likely to develop anemia. Stunted growth of children was not associated with National Health Insurance Scheme Membership. Health Education on the registration and the use of the National Health Insurance should be made a national priority to enable the Ministry of Health achieve routine Immunization targets and to reduce to the bearers minimum prevalence of anemia.

## Background

The health of children depends partially on their access to health care services. Despite the improved health care for Ghanaian children in this century as a result of reduction in the spread of infectious and contagious diseases, recent economic and social changes have called attention to new challenges to child’s health care and the need for health services. Changes in family structure, geographic mobility, and economic well-being have placed many children in need of health services resulting from conditions relating to hunger, poor housing conditions, violence, and neglect. According to Ghana Demographic and Health Survey (2008) report, 78% of children ages 6–59 months are anemic, 28% stunting and 90.6% fully immunized by card. Since many outbreaks of infectious diseases in the Western world have been blamed on incomplete immunization coverage, we can assume that childhood immunization programs help protect against infectious diseases [1]. Research evaluating the relative importance of factors that cause incomplete immunization coverage, including socioeconomic factors, parental awareness, health care provider practices, and inadequate health education and communication [2, 3], has important policy implications.

Malaria, anemia, and stunting as a result of malnutrition contribute substantially to childhood morbidity in sub-Saharan Africa. In Ghana, severe anemia results in 554 deaths in the emergency rooms per annum in children [4]. Despite evidence of the effectiveness of nutritional interventions in improving feeding practices and preventing under nutrition and anemia in sub-Sahara Africa, infant mortality still persist [5] and is a stumbling block to the achievement of the millennium development goals. The Ghana health service (2013) report of increase immunization coverage although consistently, annual immunization targets are not met. This results in the series of mob up programs organized to shore up immunization coverage to enable missed children achieved full immunization.

Studies [6, 7] have been done to examine the impact of the NHIS, the second study found that pregnant women who participated in the scheme enjoyed reduced incidence of birth complications and were more likely to receive prenatal care, deliver at a hospital, and be attended to by a trained health professional during birth. The first was just a quantitative investigation on the national health insurance scheme NHIS in Dormaa municipality, a district in Ghana. It examined why some residents remained uninsured, since its implementation has been a little over a decade now. Also a randomized control trial by [8] was not detailed on the effect of socio demographic status on child health service and utilization outcomes. There is therefore a limited statistical evidence of the association between NHIS membership and child health service outcomes and utilization. An important related issue is whether enrollment in National Health Insurance provides adequate financial protection, increases utilization of healthcare services and, ultimately, improves health outcomes.

This study analyzed data based on nationally representative survey and therefore results and conclusion could be generalized to the entire Ghanaian populace. The authors seeks to determine the association between NHIS membership, socio-economic status, geographic location and other relevant background factors, on child health service utilization and outcomes. The outcomes of interest in the study are full immunization, anemia and stunted growth in children.

## Methods

### Data source

Data from the Multiple Indicator Cluster Survey 2011(Secondary data) which is a nationally representative household sample survey of 12,150 households in 810 enumeration areas (EAs) was used. A total of 7626 under-five children were in the survey. Thus children under-five data obtained from Multiple Indicator Cluster Survey dataset was used for statistical analysis. The survey provided estimates of all key health indicators at the national and regional levels, as well as for urban and rural areas. Moreover, four of the 10 regions that are of particular importance for UNICEF’s programs were disproportionally oversampled so as to provide some data at the district level. The four over sampled regions were Central, Northern, Upper East, and Upper West [9].

### Survey design

Multiple Indicator Cluster Survey (MICS) conducted in 2011 was used. The MICS 2011 used a multi-stage stratified cluster sampling for the selection of the survey sample where the list of enumeration areas (EAs) from the 2010 Ghana Population and Housing Census (PHC) served as a frame for the MICS sample. The frame was first stratified into the 10 administrative regions in the country, then into urban and rural EAs [9]. Thus, the first stage sampling units consisted of census enumeration areas and the second stage sampling consisted of households. Within each household, all women aged 15–49 years, all under-five children and all men aged 15–49 are included in the survey. Random systematic sampling procedure was used in selecting both the enumeration areas and the households for the survey.

### Variable definition

The three main outcome variables of interest to the study include: child health service utilization (full immunization) and child health outcome (stunting and anemia). Child Health service utilization (full immunization) against common childhood illnesses (i.e. from Bacille Calmette Guérin (BCG) Vaccine to Yellow fever) according to vaccination card. Full immunization was clearly defined as all children aged between 12–23 months who had received all vaccination at the time of interview per the Ghana Health Service regulations. The six childhood killer diseases of which children are vaccinated against in Ghana are poliomyelitis, measles, diphtheria, tuberculosis, influenza, and yellow fever. Since there was no variable in the dataset representing full immunization, we created a new variable where those who had received all vaccination at the time of interview were coded as one representing full immunization and zero representing not fully immunized.

The WHO standard definition of stunting (height for age z-score below −2 standard deviation of reference population indicator for long term nutritional deprivation) was used to categorize children into stunted growth and not stunted growth.

Anemia in children was defined as hemoglobin level below or equal to10.9 g/dL. In the multivariate analysis, we first analyzed hemoglobin level as a continuous outcome to examine the how consistent the results will be when categorized as binary outcome below or equal to 10.9 g/dL.

National Health Insurance Scheme Membership was the main exposure variable measured as NHIS valid card of the child seen and valid card not seen or missing at the time of interview. The confounding variables and effects modifiers identified from literature were socio-economic status, mother’s education level, sex, age, ethnicity, religion and geographic location (Regions). These factors were obtained by combining what other published articles assumed to be confounders on the relationship between NHIS, health outcomes and service utilization. Multiple Indicator Cluster surveys employed WHO standard techniques for measuring SES, height, weight and anaemia.

### Statistical analysis

The distribution of each of variable was examined to gain an understanding of the characteristics of the study population, to check for possible outliers and what statistical technique to use. Normality and equal variance assumption of all numerical outcomes were tested using and Shapiro-Wilk and Bartlett test respectively. Graphical illustrations of continuous variable were presented with histogram, scatter plots and box plot and line graphs. For categorical variables, we used multiple bar charts.

Descriptive analysis of continuous and categorical variables were estimated using averages/means and proportions respectively. Median and interquartile range were reported instead of mean and standard deviations in situations where the variables of interest are not normally distributed. Univariate and bivariate analysis between exposure-outcome variables of interest ignoring all other variables were conducted to determine crude association. NHIS membership of children and relevant health outcomes of interest in the study were determined using Chi-Square test of association. However, because differences in the measures of utilization and child health outcomes may be affected by variables other than National Health Insurance membership, multivariate analyses was conducted. The multivariate analysis was based on Binary Logistic Regression Models and linear regression techniques to control for the potential confounding effects of the child's age, sex, socio-economic status, region, area of residence (rural/urban), and several variables deemed as potential confounders from literature. Since the main objective was to provide a valid measure of effect between NHIS membership (main exposure variable) and the two main outcome variables adjusted for several independent variables, we did not assess interaction effects of these variables. Variable selected in the final model was based on hierarchical backward selection. We then scrutinized collection of best models based on Hosmer-Lemeshow Goodness-Of-Fit (HL-GOF) as one criterion of fit and the Akaike Information Criterion (AIC). We chose *P* = 0.25 as the threshold for including variables in the multivariate model because this has been recommended elsewhere as an appropriate boundary [4]. Unless otherwise stated, only differences that were significant at the level of 0.05 (in a two-tailed test) were discussed.

Data was analyzed using Stata MP Version 11, a statistical-analysis program that incorporates the complex survey design as used in the MICS. Since complex survey design effect are essential in estimating the precision of survey estimates, child’s sampling weight was used in analyzing the under-five data and variances were estimated using Taylor Linearized variance estimation approach in a manner that reflects the complex survey design. In incorporating the design features into Stata for the analysis, enumeration areas were used as our Primary Sampling Unit (PSU) or cluster. Stratification was done with regions/rural and urban as stratum identifier. Finite Population Correction (FPC) was ignored as number in each enumeration area and households were not found in the dataset. We assume no post-stratification as the variable representing the number of households on the sampling frame for each region (rural/urban) of the country was not found in the dataset.

In checking for errors, we examine the distribution of each of the variables. For categorical variables, we checked that all observations relate to allowed categories, and that the frequencies in each category make sense. For numerical variables, range checks were performed to search for values falling outside the expected range. Histograms were also used to look for outliers that look extreme relative to the rest of the data. We conducted consistency checks, to search for cases where two or more variables are inconsistent. For example, full immunization and Poliomyelitis vaccine status were recorded; a cross-classification of the two was used to check that there was a value recorded for children vaccinated against Poliomyelitis vaccine and full immunization. Scatter plots was useful for checking the consistency of numerical variables, for example of height of child against age in months, or weight against height. For variables (ages, heights and weights) of children under five) with missing information such as hemoglobin level of children and stunting, missing value imputation techniques was used. The proportion of missing data was 4%.

## Results

The results of the study was divided into two parts namely the descriptive and the multivariate analysis of outcome variable of interest.

### Descriptive analysis of exposures and outcome variables

Table 1 shows the crude association between socio-demographic factors and valid NHIS membership. A total of 7550 households with 7626 children under-five years of age were used for the study. The proportion of males to females was 49.8% and 50.2% respectively. The median age of the children was 30 months with 29 months as the interquartile range. The proportions of respondents sampled from each region were almost identical with the exception of Upper East and Upper West which had 4.3% and 3.0% respectively. Akan’s as an ethnic group formed 18.9% of the households sampled for the study. More than half (56.5%) of the households were from the urban areas and 43.5% were from the rural areas. In relation to mothers education, 32.5% [95% CI: 30.2-34.9] had no education at all, 11.8% [95% CI: 10.3-13.5] had secondary to tertiary education. The proportion of children with NHIS membership was 86.2% [95% CI: 80.1-90.6].The proportion of households in the richest and poorest quintile was 17.4% and 22.9% respectively. For vaccination coverage, 82.4% [95% CI: 78.9-85.4] children had ever been given Yellow Fever Vaccination, 84.9% Measles, 91.7% Pentavalent, 96.6% Polio, and Bacille Calmette Guérin (BCG) Vaccine (95.8%). The vaccination on card for children seen at the time of interview was 80.9% [95%CI: 79.4-82.4]. The proportion of children ever had vaccination on card was 45.5%. Children who received full immunization between the ages of 12 months and 23 months were 77.1% [95% CI: 73.5-80.3%]. Almost all (93%) had not received any other vaccination. The prevalence of immunization among children with valid NHIS membership card seen at time of interview was 34.3%. The prevalence of anaemia at the time of interview was 54.7% [95% CI: 52.3-57.1]. The prevalence of anaemia among children with valid NHIS membership was 45.5%. The prevalence of stunting among children with valid NHIS membership was 20.8%. The median height for age z-score used to determine stunted growth was −1.2 with interquartile range of 1.7. Stunted children were 22.1% [95% CI: 20.6-23.7]. About 75% of the children had height for age z-score less than −0.35. The median and the interquartile haemoglobin level were 10.8 g/dL and 11.7 g/dL respectively. The 75th percentile haemoglobin level was 2.1 g/dL. Children with valid NHIS card was 27.1% [95% CI: 25.0-29.3].

**Table 1:** **Cross**-**tabulation of socio**-**demographic characteristics and National Health Insurance Scheme Membership of children under**-**five based on Chi**-**square test statistic with corresponding p**-**value in Ghana**, **2011**

		Total	NHIS status		Chi2; p-value
Variable			Valid NHIS membership	Not valid NHIS membership	
		N	(% 95% CI)	(% 95% CI)	
Mother's education					
	None	2455	24.0 (21.1-27.2)	76.0 (72.8-78.9)	
	Primary	1628	24.8 (21.1-28.9)	75.0 ( 71.1-78.9)	6.43; P < 0.01
	Middle/JSS	2578	28.0 (24.4-31.8)	72.0 (68.2-75.6)	
	Secondary/tertiary	889	37.0 (31.9-42.3)	63.0 (57.7-68.1)	
Residence					
	Urban	3283	29.0 (25.7-32.6)	71.0 (67.4-74.3)	
	Rural	4267	25.6 (22.9-28.4)	74.4 (71.6-77.1)	2.38; P > 0.05
Child's age (months)					
	0-11	1542	12.3 (9.9-15.0)	87.7 (85.0-90.1)	
	12-23	1453	30.8 (27.2-34.8)	69.2 (65.2-72.9)	
	24-35	1553	31.6(27.4-36.3)	68.4 (63.7-72.7)	22.35; P < 0.001
	36-47	1576	30.7 (26.7-35.0)	69.3 (65.0-73.3)	
	48-59	1426	30.2 (26.7(33.9)	69.8 (66.1-73.3	
Sex of child					
	Male	3757	26.7 (24.1-29.6)	73.3 (70.5-75.9)	
	Female	3793	27.4 (24.8-30.2)	72.6 (69.8-75.3)	0.14; P > 0.05
Religion					
	Christian	4910	27.9(25.5-30.5)	72.1 (69.5-74.6)	
	Moslem	1495	27.2 (23.1-31.6)	72.8 (68.4-76.9)	
	Traditional	455	19.6 (15.7-24.3)	80.4 (75.7-84.4)	1.715; P > 0.05
	other religion	140	14.2 (7.1-26.4)	85.8 (73.6-92.9)	
	No religion	550	28.6 (21.8-36.5)	71.3(63.5-78.3)	
Socioeconomic status					
	Poorest	1730	20.5 (17.8-23.6)	79.5 (76.5-82.2)	
	Poorer	1551	23.8 (20.2-27.9)	76.2 (72.2-79.8)	
	Poor	1559	26.4 (21.3-32.2)	73.6 (67.8-78.7)	7.27; P < 0.001
	Less poor	1397	34.5 (29.9-39.4)	65.5 (60.6-70.1)	
	Least poor	1313	32.4 (27.9-37.1)	67.7 (62.9-72.1)	

### Multivariate analysis of outcome variables

There was no association between NHIS membership and stunted growth in children [Table 2]. Factors associated with stunted growth in children included socioeconomic status, area of residence (urban and rural) and whether the child had ever been given Poliomyelitis and Pentavalent vaccine. Controlling for Poliomyelitis vaccine and socioeconomic status, children who have never received Pentavalent Vaccine are 1.4 [95% CI: 1.1-1.9] times more likely to have stunted growth than children who have received. The proportion of stunted growth attributed to children who have ever received Pentavalent vaccine to children without Pentavalent is 21.7% and 22.2% respectively. Children in the richest quintile are 16% [95% CI: 7.2-38.7] times less likely than children in the poorest quintile in developing stunted growth. Only 11.1% of the children in the rich quintile as compared to 34.0% of the children in the poor quintile had stunted growth. Children who have had Poliomyelitis vaccine are 55% [95%CI: 38.2-81.2] times less likely to have stunted growth than who do not. Since the equal variance assumption required for analysis of variance was violated, we opted for Kruskal Wallis and Wilcoxon rank sum test to determine significant difference among the quintiles. The results showed that at least two of the mean height for age z-score (WHO) which represents stunting among socioeconomic status differ.

**Table 2:** **Comparing differences between crude analysis** (**simple logistic regression**) **and adjusted analysis** (**multiple logistic regression**) **on factors associated with stunted growth of children under**-**five in Ghana**, **2011**

	Crude analysis		Adjusted analysis	
Covariates/exposure	OR (95% CI)	p-value	OR (95%)	p-value
NHIS membership (main exposure variable)				
No	1	1	1	0.200
Yes	0.90 (0.72-1.11)	0.331	0.87 (0.70-1.08)	
Sex				
Male	1	1	1	0.000^***^
Female	0.76 (0.65-0.89)	0.001^**^	0.75 (0.64-0.88)	
Age in months				
Mothers education				
None	1	1	1	1
Primary	0.82 (0.65-1.04)	0.000^***^	1.00 (0.80-1.28)	0.944
Secondary	0.54 (0.45-0.66)		0.81 (0.65-1.00)	
Tertiary	0.36 (0.25-0.50)		0.78 (0.51-1.22)	
Geographic location				
Rural	1	0.000^***^	0.97 (0.75-1.24)	0.783
Urban	1.61 (1.32-1.96)			
Socioeconomic status				
Poorest	1	0.000^***^	0.80 (0.64-0.99)	0.000^***^
Second	0.75 (0.62-0.92)		0.66 (0.50-0.87)	
Middle	0.61 (0.47-0.79)		0.42 (0.82-0.61)	
Fourth	0.39 (0.28-0.54)		0.32 (0.20-0.50)	
Richest	0.26 (0.19-0.36)			

*p < 0.05, **p < 0.01, ***p < 0.001 significant levels.

The risk factors associated with anemia included NHIS membership, socioeconomic status, stunted growth and age of the child [Table 3]. Controlling for confounding effect of socioeconomic status, age, mothers education level and geographic location, the odds of a child developing anemia for children with NHIS membership is 65.2% [95%CI: 52.9-80.2] times less than children without NHIS membership. The proportion of anemia among children with NHIS membership card is 45.5% compared to 58.6% children without NHIS membership card. Comparing the rich to the poor, it was evident that, 72.5% children in the poorest quintile developed anemia as opposed to 35.1% in the richest quintile. Anemia in children mostly occurred in the first 2 years 67% [95%CI: 55.2-71.4]. There was a significant difference in median hemoglobin level among the five wealth quintiles. The Wilcoxon rank sum test showed a significant difference in the median hemoglobin level between children in the poorest and the richest quintile (9.9 g/dL: 11.3 g/dL).

**Table 3:** **Comparing differences between crude analysis** (**simple logistic regression**) **and adjusted analysis** (**multiple logistic regression**) **on factors associated with anaemic children under**-**five**, **in Ghana**, **2011**

	Crude analysis		Adjusted analysis	
Covariates/exposure	OR (95% CI)	p-value	OR (95%)	p-value
NHIS membership (main exposure variable)				
No	1	1	1	0.000^***^
Yes	0.59 (0.49-0.72)	0.000^***^	0.36 (0.23-0.55)	
Sex				
Male	1	1	1	0.286
Female	0.74 (0.63-0.88)	0.001^**^	0.79 (0.51-1.22)	
Age in months				
6–11	1	0.034^*^	1	0.638
12–23	1.25 (0.91-1.71)		0.69 (0.15-0.19)	
24–35	0.70 (0.50-0.97)		0.51 (0.12-2.16)	
36–47	0.62 (0.48-0.84)		0.51 (0.13-2.06)	
48-59	0.51 (0.36-0.71)		0.30 (0.07-1.28)	
Mothers education				
None	1	0.000^***^	1	0.02*
Primary	0.70 (0.55-0.89)		0.83 (0.63-1.10)	
Secondary	0.45 (0.36-0.57)		0.64 (0.49-0.83)	
Tertiary	0.33 (0.24-0.47)		0.68 (0.45-1.02)	
Geographic location				
Rural	1	1	1	0.396
Urban	0.53 (0.46-0.65)	0.000^***^	0.79 (0.47-1.35)	
Socioeconomic status				
Poorest	1	0.000^***^	1	0.000^***^
Second	0.63 (0.49-0.18)		0.66 (0.37-1.18)	
Middle	0.46 (0.34-0.63)		0.37 (0.20-0.69)	
Fourth	0.33 (0.24-0.45)		0.39 (0.20-0.76)	
Richest	0.21 (0.15-0.28)		0.13 (0.07-0.25)	

*p < 0.05, **p < 0.01, ***p < 0.001 significant levels.

Full immunization against common childhood illnesses was found to be associated with NHIS membership [Table 4]. Controlling for the confounding effects of age, socioeconomic status, mothers education level, and geographic location, the odds of being fully immunized against common childhood illnesses for children with NHIS membership is 2.3 [95% CI: 1.4-3.7] times higher than children without NHIS membership. The other factors associated with full immunization included mothers education level and geographic location.

**Table 4:** **Comparing differences between crude analysis** (**simple logistic regression**) **and adjusted analysis** (**multiple logistic regression**) **on factors associated with Full Immunization of children under**-**five in Ghana**, **2011**

	Crude analysis		Adjusted analysis	
Covariates/exposure	OR (95% CI)	p-value	OR (95%)	p-value
NHIS membership (main exposure variable)				
No	1	1	1	0.002^**^
Yes	2.30 (1.42-3.72)	0.000^***^	2.15 (1.34-3.47)	
Sex				
Male	1	1	1	0.265
Female	1.21 (0.84-1.74)	0.306	1.24 (0.85-1.79)	
Age in months	1.01 (0.96-1.07)	0.661	1.01 (0.96-1.07)	0.647
Mothers education				
None	1	1	1	0.696
Primary	0.91 (0.56-1.47)	0.703	0.90 (0.52-1.55)	
Secondary	1.47 (0.94-2.31)		1.51 (0.87-2.62)	
Tertiary	1.83 (0.96-3.39)		1.83 (0.81-4.14)	
Geographic location				
Rural	1	1	1	0.020^*^
Urban	1.32 (0.89-1.96)	0.161	1.86 (1.10-3.12)	
Socioeconomic status				
Poorest	1	0.710	0.91 (0.54-1.53)	0.715
Second	0.91(0.56-1.49)		1.03 (0.52-2.03)	
Middle	1.02 (0.60-1.71)		1.61 (0.72-3.59)	
Fourth	1.41 (0.79-2.53)		1.26 (0.50-3.16)	
Richest	1.25 (0.68-2.30)			

*p < 0.05, **p < 0.01, ***p < 0.001 significant levels.

## Discussion

This paper investigated the correlation between NHIS insurance enrolment, socio-economic and demographic status on child health service and utilization outcomes.

Issues have been raised regarding equity and the sustainability of the NHIS scheme. The main equity concern is that wealthier Ghanaians are currently more likely to enroll than poorer Ghanaians, despite premiums that were supposed to vary by income, but in practice are fixed at one level for all in general, and premium exemptions for the “core poor” [10]. This was evident in this study as wealth index quintiles correlated with NHIS membership of the children and with Health Systems 20/20 Project and Ansah et al. [8].

Our study also identified mother’s education level and geographic location to be associated with immunization. According to Lieu et al. [11], financing reform may improve immunization delivery and reduce the load on public clinics [11]. However, legislation to improve immunization financing will not achieve optimal results unless parent education is improved and organizational barriers are also removed. Rodewald et al. [12] showed that insurance coverage for low-income working families resulted in a shift in the provision of immunizations from the health department to primary care providers and this increased immunization coverage [12], thus result of this study confirms the results obtained in various studies. In a cross-sectional survey on factors influencing immunization coverage in Sudan, the authors found that children in urban and rural areas differed significantly in their reported vaccination coverage. In urban areas, accessibility to immunization centers is high compared to rural areas where amidst the few centers where immunization is schedule based [13]. Schoeps et al. [14] noted that children living in rural areas were more likely to fail timely vaccination with BCG than urban children [14]. Brown and Oluwatosin [15] also identified that children in urban areas were more likely to complete immunization [15]. Contrary to these findings, Etana and Deresa [16] found that area of residence and mother's socio-demographic characteristics were not significantly associated with full immunization among children [16]. Topuzoglu et al. [17] indicated that barriers to immunization services were related to the economic constraints and accessibility of the services [17]. This clearly indicates that poor people find it difficult to pay for insurance premium and hence their inability to access full immunization.

It is well understood from other literature [18] that Culture, religion and ethnicity have a broad influence on beliefs and practices related to health, illness and healing. This influence includes definitions of health and illness, beliefs about the causes of health and illness, decisions about whether or not to seek formal health care, and decisions about the type of health care provider to be sought. In consonance with the findings from this study, it was realized that traditionalist were less likely of registering for the NHIS as compared to Christians and other religious groups. However, in this study, ethnicity had no influence on decision to enroll in the NHIS.

The Ministry of Health, Ghana report that child health is a major public health and development challenge in Ghana [19]. In this recent study, there was the less likelihood of children with mothers on NHIS to develop anemia as compared with those not on NHIS. Until 2008 there has been a 30% reduction in the under-five mortality rate due to anemia [19]. It is therefore refreshing that these studies have associated increase in a form of free health care services among under-fives to the decrease in anemia in these age groups. This is partly due to easy access of the mother to healthcare in recent times, an improvement on figures from the Ghana Health service in 2003 which indicated that about 75% of children in Ghana were having some degree of anemia, and was attributed to cost.

Our sample analysis did not show correlation between NHIS membership and stunted growth in children [Table 3]. This partly confirmed the results obtained by Ansah and Narh-Bana [8] where they concluded that removing out-of-pocket payments for health care had an impact on health care-seeking behavior but not on the health outcomes measured. Our findings however seemed to contrast two different studies. A study conducted by Binagwaho and Hartwig showed that insurance scheme seems to have contributed to improvement in stunting and mortality, at the critical stages (before the age of two) [20]. Imai [20] also concluded that access to health insurance and health related facility, infrastructure and environments are important factors in reducing child malnutrition that contribute to stunted growth in children [21].

Regional variation was also considerable with children from the Northern Region being almost three times more likely to be stunted than those from the Greater Accra region. As expected, older children were substantially more likely to be stunted, given the historicity of the problem. The results obtained based on 2011 MICS data, indicated that socioeconomic status is a significant factor associated with stunting. Although NHIS correlated positively with socio economic status, in that the rich and the urban residence had the ability to pay for NHIS membership, there was no association between NHIS membership and stunted growth. This study noted that in addition to socioeconomic status, children who had ever received Poliomyelitis and the Pentavalent vaccines were less likely to be stunted. The government and other developing agencies in realizing the need for urgent intervention to curb stunting and malnutrition in general has implemented a number of priority interventions. Under the overall child survival framework of High Impact Rapid Delivery (HIRD) approach, the growing commitment for nutrition by the Ministry of Health enabled the Ghana Health Service to increase the annual coverage of two doses of vitamin A supplements to more than 80 percent. The current policy and program/project environment is favorable for prioritizing, strengthening, and coordinating nutrition and child survival programs at all levels [22].

In sub-Saharan Africa, male children under five years of age are more likely to become stunted than females, which might suggest that male children are more vulnerable to health inequalities than their female counterparts in the same age groups. A study by [23] showed that in several surveys in sub Saharan Africa, sex differences in stunting were more pronounced in the lowest Socio Economic Status groups. Male children in households of the poorest 40% were more likely to be stunted compared to females in the same group, but the pattern was not consistent in all their studies used for the Meta analysis.

This current study revealed that children with valid NHIS membership card are more likely to be fully immunized and utilize immunization programs. Although immunization of children in Ghana is independent of valid NHIS membership, there is a sizeable number of caretakers/households in Ghana who still believe that with valid NHIS membership, children could utilize or have access to all forms of healthcare including immunization. A randomized control trial conducted in rural Ghana by Ansah and Narh-Bana [8] found that children in households randomized to free healthcare used formal healthcare more and informal healthcare less than a control group. According to Chin-Shyan Chen and Tsai-Ching Liu et al. [24], by providing free well-baby care through NHIS, health planners have successfully stimulated the demand for immunization. Removing user fees led to increased health care utilisation in South Africa and Uganda as noted by these articles [25, 26].

NHIS membership was found to be associated with anemia in children under-five. Prevalence of anemia among children with NHIS membership was far less than children without valid membership. Most people with valid NHIS membership card seek treatment in various health facilities across Ghana (Private or Government hospitals) and therefore have the opportunity to seek medical advice from health practitioners on conditions related to anemia hence the reduction in proportion of anemia in those group. The results obtained correlates with the findings of Dong et al. [27] where they concluded that National Health insurance provides an assessment of anemia prevalence based on laboratory assessment. Ansah et al. [8] found that removing direct payments for health care in the public sector increased the use of primary care clinics by 20% and lowered out-of-pocket spending on health care by 37% for families of children with anemia at baseline.

The strength of the study lies in that fact that sample are from nationally representation survey and conclusions can therefore be generalized to the Ghanaian children under-five years of age. Although we have controlled for some confounding variables in the model selection, time variant and invariant characteristics that are not observed and that cannot directly be controlled for remain a concern. The decision to join a health insurance scheme is determined by unobserved factors that could simultaneously impact the health care utilization and child health outcomes.

## Conclusions

There is correlation between NHIS membership and socioeconomic status, mother’s education level, age of the child and geographic location. NHIS membership was found to be related to child health service utilization (full immunization) of children and whether a child becomes anemic. Stunted growth of children was not associated with NHIS membership but was found to be associated with socioeconomic status and therefore the need for further studies to establish what could account for the disconnection between NHIS membership, socioeconomic status and stunted growth.

All children should have health insurance independent of the socioeconomic status, mother’s education level, age of the child and geographic location as this would help mitigate anemia prevalence and ensure full immunization of children. A policy direction on women access to employment and education are crucial for promoting sustainable development. Therefore there is the need to increase education throughout the country and that access to good education will increase socioeconomic status which would in turn enhance NHIS enrolment and good child health utilization outcome. Awareness creation on the importance and benefit of the NHIS among the uneducated is crucial for the sustainability of the scheme. Also there is the need for the Ministry of Education and the Ministry of Health to work hand-in-hand for the long term sustenance of NHIS enrolment. To minimize stunted growth among children, we recommend that government should intensify the already existing strategies aimed at curbing the menace. More prudent economic policies aimed at elevating those in the lower quintile should be implemented to bridge the gap between the rich and the poor. This would enable more parents/caretakers to pay their premium to enjoy full benefits associated with NHIS membership. Steady geographical spread of NHIS coverage as seen in the study is refreshing; nevertheless policies targeted at aggressive spread of health facilities around the regions of low coverage might enhance NHIS enrolment. It is quite indicative that direct cost of care is a barrier to the poorest in accessing health care from this study, but it is not the only one, and other modifiable barriers have to be addressed if removing the direct cost of care is to have a useful impact on the health of the poorest.

### Definition

*Gold Standard odds ratio* is an estimate of odds ratio controlling for all independent variables in the model.

## Abbreviations

NHIS: National health insurance scheme
GSS: Ghana Statistical Service
EPI: Expanded program immunization
PSU: Primary sampling unit
SSU: Secondary sampling unit
MICS: Multiple Indicator Cluster Survey.
